# The Alexander Operation — Its Immediate and Remote Results

**Published:** 1905-01

**Authors:** James E. King

**Affiliations:** Buffalo, N. Y., Adjunct Professor of Obstetrics, University of Buffalo ; Attending Gynecologist, Erie County Hospital; Assistant Gynecologist, Buffalo General Hospital; Fellow British Gynecological Society, London.


### The Alexander Operation — Its Immediate and Remote Results.

By JAMES E. KING, M. D., Buffalo, N. Y.,
Adjunct Professor of Obstetrics, University of Buffalo; Attending Gynecologist, Erie County Hospital; Assistant Gynecologist, Buffalo General Hospital; Fellow British Gynecological Society, London.

MY object or, perhaps better, my apology for asking your attention to the much discussed Alexander operation, is not that I have a new technic to offer, but only to consider a few of the clinical features which are results, immediate or remote, of the operation. It is an operation that has been much criticised favorably and unfavorably, but the results of the discussion and the experience of many operators, whose cases number in the hundreds, has been to place it upon a firm foundation as one of the surgical resources of gynecology. Successful results are only possible when the indication for it is carefully observed. It is agreed by all that it never should be undertaken in the presence of any complicating condition of the uterus or adnexa unless such condition may be treated satisfactorily at the same time. This, then, reduces the indications to very simple terms—namely, simple uncomplicated retroversion which implies a most careful diagnosis. We do not expect an Alexander operation to relieve symptoms with complicating disease of uterus or adnexa and it is therefore unfair to condemn the procedure when done under such circumstances.

In the correction of simple retroversion we are dealing with tissue which is in no sense pathologic and the operation aims at a mechanical result. The correction of the retroversion is usually permanent. Cases of relapse may often be traced to suppuration or to the presence of unusually small ligaments. The latter failures should not be charged to the operation but to the unfortunate choice of the method in those particular cases. Granting that relapse may occasionally occur, it is rather from the patient's viewpoint than from the mechanical results that the few clinical features will be discussed. It is unfortunate that the surgeon and patient cannot agree always as to a successful result in operations.

One of the unpleasant sequelæ is pain. It is usually unilateral, extending up in the abdominal wall about three inches. It is sometimes quite severe and is intensified by pressure or walking. It may come on directly following the operation, or it may be delayed for a few days. It often remains four or five months, gradually improving until ultimately it entirely disappears. When it is severe, the patient may be unable to wear her corset. The cause for this pain seems to be injury to the ilio-inguinal nerve. The nerve as it passes through the inguinal canal from the abdominal muscles to its cutaneous distribution, lies in very intimate relation to the round ligament. It may be injured in its separation from the cord or in pulling the ligament out; and, finally, it may be sewed in with the fascia when the canal is closed. It is also often seen in the wounds that have suppurated, the nerve doubtless being held in the resulting scar tissue. Great care should be observed in avoiding further handling of the nerve than is necessary for its separation from the ligament, and in closing the canal the nerve should be carefully placed and not sewed in with the fascia. To the patient, the pain is often very troublesome and unpleasant and, unfortunately, there is little that can be done for its relief.

Another result which is sometimes seen, and which is far from satisfactory to the patient or surgeon, is the failure of the corrected retroversion to relieve all the symptoms. The fundus may be well forward and held so, but the patient is conscious of a feeling of dragging and weight in the pelvis. Nearly all cases of retroversion are accompanied by some relaxation of the uterosacral ligaments and consequent descent of the uterus, depending upon the amount of relaxation and the condition of the pelvic floor. In some cases the symptoms seem largely to be due to the condition of these uterosacral ligaments. Two cases of my own will best illustrate these observations.

Mrs. B., age, 27; married five years; three years ago labor was induced at seven months for dangerous kidney symptoms; eighteen months later she became pregnant again, but aborted at four months, the fetus presenting the appearance of having been dead about ten days. When first I saw the patient she described symptoms of retroversion, the history indicating its existence for some time before marriage. Examination showed a freely movable displaced uterus. No prolapsed ovary could be felt nor was there evidence of uterine or adnexal disease. The uterosacral ligaments were much relaxed, allowing descent of uterus. Examination at the time of operation verified previous findings. The Alexander operation was done, the ligaments being found of good size. Following the operation a pessary was worn for three months, during which time the patient was quite free from unpleasant pelvic symptoms. A short time after removal of the pessary, symptoms of weight and dragging in the pelvis were complained of. Examination showed fundus of uterus well forward and normal in size; ovaries apparently in position. There was, however, still marked relaxation of uterosacral ligaments and descent of cervix. A pessary was again placed with the relief of symptoms. Removal of the pessary was each time soon followed by recurrence of symptoms, so that for a year and a half the pessary has been necessary for the patient's comfort.

The second case is Miss M., age, 22. She gave the usual history of retroversion and dysmenorrhea of four years standing. Examination disclosed a freely movable retroverted uterus, with no adnexal disease, but marked relaxation of uterosacral ligaments. Examination under ether at the time of operation gave the same results. The Alexander operation was done and a pessary worn for two months following operative intervention. Three weeks after its removal the patient returned, complaining of backache with dragging and weight in the pelvis. The fundus of the uterus was found well forward, but the cervix was low, due to the relaxation of uterosacral ligaments. A pessary was fitted with complete relief. For six months past she has worn the pessary and has made no complaint.

These two cases may be regarded as a type of the "successful operation" that does not cure the patient. That the symptoms in both cases were due to the relaxation of the uterosacral ligaments seems conclusive, inasmuch as refitting a pessary afforded relief. Such a sequence does not, however, always follow in the presence of relaxation of these ligaments, for in many cases in which there is marked descent the symptoms are completely relieved by the operation alone. It is simply a difficulty which results from the lack of uniformity in the symptoms produced by the same condition in different individuals.

The appearance of hernia following the operation is sometimes seen. Many such cases have been collected. Faulty technic and suppuration are in nine cases out of ten often responsible for it. The same care should be observed in sewing up the canal as in the radical operation for the cure of hernia. When so done hernia must be a rare sequel. Suppuration should never occur. The technic in sewing up the canals and treatment of the ligaments must be modified somewhat by the size of the cord and extent to which the canal has been opened.

The operation is admitted to have no influence upon pregnancy or labor and, so far as my experience goes, pregnancy and labor do not cause recurrence of the displacement. I have attended five women in labors occurring at variable lengths of time following the operation. In two, the operations were performed by other operators; three cases were my own. Four of the five women I have been able to observe after puerperium. In each case a pessary was fitted as a precautionary measure and worn for two months during the period to complete involution. In none of these cases has there been any return of the displacement. One patient, during the early weeks of pregnancy, complained of "pulling" at the side of the wound sites, which was relieved by support until the uterus became large enough to rise out of the pelvis.

To gather statistics to determine what percentage of recurrence follows the Alexander operation is rather uncertain in its results. In hospital work it is impossible to follow many of the cases we operate upon, but I believe that recurrence should be very rare if the technic of the operation is varied according to what, in the judgment of the surgeon, seems to be the requirements of the case as determined by the size of the ligaments. When all is said and done it must be admitted that the Alexander operation has a prominent place in the treatment of uncomplicated retroversion, and reference to my case book will bear evidence of many patients who may say, with the surgeon, that the operation is a "perfect success."

93 Niagara Street.

